# Managing Distress Over Time in Psychotherapy: Guiding the Client in and Through Intense Emotional Work

**DOI:** 10.3389/fpsyg.2019.03052

**Published:** 2020-02-19

**Authors:** Peter Muntigl

**Affiliations:** Faculty of Education, Simon Fraser University, Burnaby, BC, Canada

**Keywords:** affectual stance, affiliation, client-centered therapy, conversation analysis, crying, distress, emotion, empathy

## Abstract

Clients who seek psychotherapeutic treatment have had personal experiences involving some form of distress. Although research has shown that the client's ability to experience and express painful emotions during therapy can have a therapeutic benefit, it has also been argued that displaying distress may convey a form of helplessness and vulnerability, and thus, clients may be reluctant to cast themselves in this light. Using the methods of conversation analysis, this paper explores how a client's upsetting experience is managed over the course of a single session of client-centered therapy. The main analytic focus will be on (1) the different therapist practices used to orient to the client's distress, (2) the varying forms of client opposition to the therapist's attempts to work with the distress, and (3) the context sensitivity of orienting to distress and how certain practices may be uniquely shaped by what had occurred in prior talk. It was found that, whereas certain types of therapist responses tended to be endorsed by the client, others were forcefully rejected as inappropriate displays of understanding or empathy. By focusing on repeated sequential episodes over time in which a client conveys distress, followed by the therapist's response, this paper sheds light on the interactional trajectory through which a client and therapist are able to resolve impasses to emotional exploration and to successfully secure extended and intense emotional work.

## Introduction

Psychotherapy offers a setting in which clients are able to report on their personal experiences, some of which involve intense moments of distress. These contexts of self-disclosure are believed to have positive therapeutic benefit. According to Greenberg et al. (1993, p. 271), “some of the most powerful moments in therapy occur when clients allow themselves to experience and express extremely painful self-relevant emotions.” Notwithstanding the immense potential value of emotional self-disclosure for facilitating productive therapeutic work, conveying upsetting personal experiences also creates certain interactional challenges. The first relates to the difficulty that therapists may have in responding to the client's past or present feelings in an appropriately congruent manner; that is, therapist responses may not necessarily fit with clients' understandings of their distress, thus infringing on the troubles teller's ownership of personal experience (Sacks, 1995b). Heritage (2011) has termed this challenge a *problem of experience*. Second, clients may not only report on their past distressing experience but may also simultaneously express upset in the present moment (e.g., crying). Thus, in choosing to affiliate with the client's distress, therapists may not only need to decide which aspect of the distress (i.e., what is reported or expressed) should be oriented to first but may also need to manage distress at both these levels.

These challenges surrounding the client's personal experience of distress are a central concern in psychotherapeutic interactions. If they are dealt with successfully, the therapist and client may strengthen their relationship by creating *communicative attunement* (Elliott et al., 2011) or an *empathic moment* (Heritage, 2011) in which they display shared understanding and mutual affiliation within a sequence of talk. If, however, the management of these concerns is less successful, as when the client rejects the therapist's display of empathy or the therapist does not affiliate in the “appropriate” way with the client's distress, tension and discord may arise in the relationship, and further, the local therapeutic goal of guiding clients through their experiences of grief may be in danger of becoming derailed.

Using the methods of conversation analysis (CA) (Sidnell and Stivers, 2013), this paper examines how a client's upsetting experience is managed over the course of a single session of client-centered therapy. By focusing on repeated sequential episodes over time in which a client conveys distress, followed by the therapist's response, it will be shown (1) how the therapist orients to the client's upset in different ways, (2) how the client opposes the therapist's attempts to work with the distress, and (3) how these disaffiliative sequences provide a novel context in which the therapist can orient in an alternative way to the client's emotional experience. It was found that, whereas certain response types are endorsed by the client, others are forcefully rejected as inappropriate displays of understanding or empathy. It is the latter client responses that draw specific attention to the “problem of experience” and, further, mandate a subsequent reaffiliative move from the therapist. The focus is also placed on how the client and therapist orient to the client's vulnerability in these moments of upset and, further, how the client may use “vulnerability” as a resource to resist further exploration of her feelings in the present moment of therapy. Finally, this single case analysis is illustrative of how, at the end of the episode, the client and therapist are able to resolve impasses to emotional exploration. This case, therefore, maps out the productive—and clinically relevant—trajectory through which a client and therapist are able to successfully secure extended and intense emotional work.

## Dilemmas of Personal Experience

One of the guiding principles behind client-centered therapy is the provision of empathy by privileging and validating the client's ownership of experience (Rogers, 1951). When adopting an empathic stance, Rogers (1957) recommends that therapists also appear *genuine* or *authentic* and show *positive regard* toward the client. Whereas, genuineness means relating to the client's experience in a transparent manner, without putting on a professional attitude or facade that is incongruent to the client's needs (Lietaer, 1993), positive regard refers to “prizing the person” or displaying unconditional acceptance of the client's feelings and experience. Thus, when responding to clients' reports of experience, therapists need to find the right balance between these elements to do productive relationship work. There is certainly a heightened awareness within psychotherapy that offering the appropriate kind of empathy, for example, may pose a significant challenge in certain contexts. As Elliott et al. (2011) have argued, therapists may sometimes need to individualize their response to best suit their client and to know when empathy is called for and when it is not; for example, they have noted that clients who communicate their “inner experiences” more openly may respond favorably to various forms of empathic displays, whereas clients who are “fragile” may instead show an adverse reaction.

By reporting on significant and often distressing episodes of their lives, clients provide therapists with detailed access to their emotions and assessments, or *affectual stance* (Stivers, 2008), pertaining to persons and events. Because reported experiences are infused with affect, they help to build up and create the necessary materials or resources through which therapists may offer affiliation or empathy and, moreover, strengthen the therapist/client relationship. For this paper, empathic responses are viewed as social actions that endorse and display understanding of the teller's felt experience (Heritage, 2011; Kupetz, 2014; Muntigl et al., 2014), in such a way as to ratify the teller's epistemic authority through a range of epistemic markers that index contingency (Hepburn and Potter, 2007).

When persons report to others about their personal experiences, two moral systems become relevant for the interaction (Heritage, 2011). The first is that these disclosures are considered to be “owned” by the experiencer and thus index specific entitlements that are associated with having experienced something first hand (Pomerantz, 1980; Sacks, 1995b): primary rights to know about what happened and to react emotionally or develop an elaborate affectual stance to the event in question. The second is that, in sharing personal experience with others, recipients are mandated to display empathy with the teller's experience. In Heritage's (2011) view, these moral systems may *collide* and cause tension, especially when the empathic response is seen as inappropriate and as infringing on the teller's ownership of experience. Thus, to ensure that an affiliative episode can be achieved, recipients must successfully attend to these relevant interactional issues or dilemmas.

## Affiliating With Distress Displays

Psychotherapy researchers have noted that distress displays may index client vulnerability in which clients may experience themselves as helpless and lacking control (Greenberg et al., 1993; Greenberg and Paivio, 1997). In these contexts, client-centered therapists face a formidable challenge. On the one hand, therapists are mandated to validate and show understanding of the client's distress. However, on the other hand, clients may resist further topicalization of the distress and further talk that draws even more attention to their vulnerability or helplessness. Expressions of distress may also be seen as opportunities to engage more directly with what is upsetting the client in the present moment of therapy. Drawing attention to the client's emotional experience in the here and now of therapy is considered to be an effective and beneficial practice in many therapeutic approaches (Rogers, 1959; Perls, 1973; Bugental, 1999; Yalom, 2002; Stern, 2004; Kondratyuk and Perakyla, 2011). Within client-centered therapy, for instance, Rogers (1959, p. 198) argues that client utterances making reference to present moment experience (e.g., “for the first time, right now, I feel that you like me”) are referred to as “experiencing a feeling fully, in the immediate present. The individual is then congruent in his experience (of the feeling), his awareness (of it), and his expression (of it).” However, here also, clients may be reluctant to engage more deeply with and express their anguish in the present moment for fear of being too exposed or vulnerable. The difficulty for therapists, therefore, is to offer clients enough security through which they may risk more directly confronting their upsetting experience.

Within CA, there is a growing interest in examining how distress is interactionally dealt with in a variety of institutional contexts, such as caller help lines (Hepburn and Potter, 2007, 2012), medical encounters (Beach and Dixson, 2001), and police interviews (Antaki et al., 2015). One commonly identified response to distress displays that communicates a high degree of empathy is *formulating*. Here, the recipient either provides the gist or summary of preceding talk or draws an implication or upshot of what had been said (Heritage and Watson, 1979; Antaki, 2008)^1^. Other practices, seen in caller help lines and police interviews, have been termed *take-your times* (Hepburn and Potter, 2007) and function to manage emotional disruptions to talk by orienting to the difficulty the distressed speaker has in completing his or her turn.

It is argued that distress may be conveyed in interaction in either of two forms: through a reporting of a past distressful event or via an *in-the-moment* expression of distress (Antaki et al., 2015). In the latter sense, distress is something that emerges in the here and now and is built up through talk and other non-verbal means. Wootton (2012, p. 43) provides a useful working definition when he characterizes *distress* “as roughly denoting those forms of tearfulness which have crying as their most extreme form of expression.” In her influential work on crying, Hepburn (2004) has shown that distress may be indexed within expressions or interactional features of varying intensity, such as sniffs, tremulous voice, and sobbing. Other possible distress markers have been noted by Hoey (2014) in reference to sighing. The distinction between reporting a distressing event and expressing distress in the moment may, however, not always be so clear cut in interaction. In psychotherapy, for example, a client's reporting of a distressing experience may be accompanied by in-the-moment distress markers (e.g., sniffs, tremulous voice, etc.). In such contexts, client-centered therapists may need to attend to one or the other forms of distress, for example, by exploring what the past feelings of distress meant (i.e., attending to the report of distress) or by exploring the client's present feelings (i.e., attending to the in-the-moment distress). Alternately, therapists may try to balance these different facets of distress by attending to them sequentially. What will be shown in this paper is how a therapist orients to these different levels of distress and how certain responses end up facilitating or delaying emotional exploration.

## Data And Methods

The case under examination was taken from the York I Depression Study (Greenberg and Watson, 1998) and forms part of a larger project that examines therapist–client affiliation and disaffiliation (Muntigl et al., 2013; Muntigl and Horvath, 2014b). The client, Eve, is female and was offered 20 sessions of treatment for depression. The therapist is also female, and her mode of practice was client centered (Rogers, 1951). All sessions were video-taped. For this investigation, written informed consent was obtained from the participant for the publication of anonymized data. Persons referred to within therapy, including the client, have been given pseudonyms. From this case, session 15 was selected for transcription and analysis because it represented an extended episode of talk—comprising approximately the first 30 min of the session—in which the therapist made repeated attempts to manage the client's distress. Within this session, Eve topicalizes her upset feelings involving her brother's death that occurred ~4 years ago. These feelings were triggered by having watched the recording of the previous week's video-taped session (together with a psychologist from the York I study) in which Eve began to discuss this painful incident. A single session, involving topically related episodes of interaction, was chosen to illustrate the important therapeutic practice of distress management (see Schegloff, 1987 for a discussion of this mode of data analysis involving single, extended episodes of interaction). Thus, the aim was to shed important light on how a salient personal experience is dealt with over time and how a therapist and client eventually work through the client's avoidance to perform more intense emotional work.

The methods of CA were used to transcribe and analyze the session. The transcription notation was based on Hepburn and Bolden (2012) and Mondada's (2016) conventions for multimodal transcription (see Table 1 for the list of transcription notations used). Because client distress and its realization was a major focus in this study, Hepburn's (2004) conventions for transcribing different features of crying were also adopted; for instance, a sniff was transcribed as “°.snih°,” and tremulous voice was represented by tildes that enclose a stretch of talk “~.” Sighing was also noted and portrayed as an in-breath “.hh” followed by an out-breath of relatively great intensity “hx” (Hoey, 2014). Because exhalations in sighing are typically high intensity and often were heard to contain a voiceless velar fricative sound, similar to German “ach,” Hoey's convention of writing “x” (or “X” for higher intensity) rather than the standard Jefferson (2004) convention of “h” was used—Sighs not containing the voiceless velar fricative sound were transcribed with “h” rather than “x.” Furthermore, accompanying visible conduct, such as shoulder and chest heaving were also noted during sighing where they occurred (Hoey, 2014; Hepburn and Bolden, 2017). Sequence organization was examined with respect to three interconnected sequential slots that typically occur in psychotherapy interaction (Peräkylä, 2019): an initiating action, followed by a responding action and ending with a *third position* action that closes the exchange. In terms of distress display sequences, initiating actions involved a display or report of distress, followed by the therapist's response to the emergence of client distress and, finally, the next position in which the client ratifies or rejects the therapist's action. In the case of rejection, the therapist's subsequent practices to restore affiliation were examined.

**Table 1:** Transcription notation.

**Symbol**	**Meaning**	**Symbol**	**Meaning**
**Transcription notation**			
[	Starting point of overlapping talk	↓word	Markedly downward shift in pitch
]	Endpoint of overlapping talk	↑word	Markedly upward shift in pitch
(1.5)	Silence measured in seconds	.hhh	Audible inhalation, # of h's indicate length
(.)	Silence <0.2-s		
.	Falling intonation at end of utterance	Hhh	Audible exhalation, # of h's indicate length
,	continuing intonation at end of utterance	heh/huh/hah/hih	Laugh particles
?	Rising intonation at end of utterance	wo(h)rd	Laugh particle/outbreath inserted within a word
(word)	Transcriber's guess		
( )	Inaudible section	.hh hx	Sigh
wor-	Truncated, cut-off speech	~word~	Tremulous/wobbly voice through text
wo:rd	Prolongation of sound	.snih	Sniff
word=word	Latching (no audible break between words)	huhh.hhihHuyuh	Sobbing
<word>	stretch of talk slower, drawn out	>hhuh<	Sobbing—produced at a faster rate
>word<	Stretch of talk rushed, compressed	↑hhuh<	Sobbing—if sharply inhaled or exhaled
°word°	Stretch of talk spoken quietly	((cough))	Audible non-speech sounds
Word	Emphasis	*(blue)*	Non-verbal behavior (actor indicated by initial)
WORD	Markedly loud		

## The Session Trajectory of Managing Client Distress

From the analysis of how the client, Eve, conveyed distress—by reporting personal upsetting experiences and/or by displaying distress in the moment—and how the client's distress talk was responded to and subsequently negotiated over extended sequences, a certain interactional trajectory involving discreet phases, roughly corresponding to a *beginning, middle*, and *end* (Sacks, 1995b; Robinson, 2013), was identified. In more functional terms, these phases may be described as (1) launching a distressing episode of personal experience: orienting to the client's vulnerability; (2) managing continued opposition to emotional exploration; and (3) successful guidance into emotional exploration. The beginning phase consisted of the client's initial reporting of her experience of having watched the prior week's video-recorded session (see Extract 1). While recounting her experience, Eve displayed distress in the present moment, during which the therapist attempted but failed to guide the client into exploring her distress concerning her brother's death more deeply. During this time, the client's vulnerability became repeatedly topicalized and was used as a resource to avoid the therapist's attempts at exploration. Eve's opposition to engage with her present emotions seemed to pave the way for the next, middle phase in which the therapist repeatedly managed the client's opposition to emotional exploration (see Extracts 2, 3). This phase of talk primarily contained a series of formulation sequences in which the therapist would focus instead on the client's reporting of distress, rather than on her here-and-now distress displays. It was found that, although the client tended to affiliate with therapist gist formulations that displayed empathy, subsequent therapist responses that drew more elaborate implications of what the client had said were forcefully resisted and strongly criticized for its inappropriateness. The therapist would work to reaffiliate with the client in two ways: first, by endorsing the client's criticism (Extract 2); then, after being repeatedly reproached, by topicalizing the therapist–client relationship and the anger that the client may have felt from having watched the video (Extract 3). Finally, the third and end phase comprised a resolution in which the client began to work with—rather than resist—therapist actions that targeted emotional expression (see Extracts 4, 5). Here, the therapist alternated between different levels of client distress by responding to the client's reported distress vs. her distress displayed in the moment. By timing her responses in this alternating fashion and by a bodily movement that created a more intimate space between the interlocutors, the therapist was able to secure an empathic moment between herself and the client, which resulted in the client being guided more deeply into immediacy (i.e., how she feels in the here and now) through the production of an elaborate and extended emotional display (Extract 4). Following the client's emotional outburst, the therapist would use directive actions to maintain the client's focus on her distress (Extract 5). These phases are illustrated in Table 2 and discussed in the remainder of the *Analysis* section.

**Table 2:** The session trajectory of managing client distress in terms of three discrete phases.

**Phase 1 Launching a distressing episode**	**Phase 2 Managing opposition to exploration**	**Phase 3 Successful guidance into emotional exploration**
Client's initial distress display Therapist's response to guide client into immediacy Client's opposition to exploring her distress Orientation to client's vulnerability	1. Therapist responses that orient to client's report of distress Summary formulations Upshot formulations 2. Client's repeated rejection and criticism of therapist's upshot formulations 3. Therapist response that topicalizes the relationship, addressing client's opposition and anger	1. Therapist responses orienting to client's in-the-moment distress/abandoning rational-focused talk Immediacy questions Noticings Bodily movement to decrease physical space between therapist–client 2. Maintaining focus on client distress Therapist directive actions

### Phase 1: Launching a Distressing Episode of Personal Experience: Orienting to the Client's Vulnerability

Just prior to Extract 1, the client, Eve, reported on her experience of having looked at the video of the prior session with a psychologist from the York 1 study. The general topic of that session involved her brother's death that occurred ~4 years ago. The first mention of this incident was not elicited by the therapist but rather was launched by the client (“it's sort of< funny after watching that video last week”). Because Eve's report appears at the very beginning of the session, it speaks to the importance and newsworthiness of the event (Sacks, 1995b). The analysis of this extract will show how the therapist first responds to the client's in-the-moment distress and how Eve, for the most part, avoids or resists the therapist's efforts by repeatedly topicalizing her vulnerability. As a result, the therapist shifts her orientation from exploring Eve's feelings in relation to having watched the video to addressing the client's vulnerability in terms of not being able to engage with her present distress.

In line 06, the client reports being “caught off guard.” This implies that something “unexpected,” in which she has no control over, had happened. At this time, her talk adopts a tremulous voice quality (see Hepburn, 2004), which signals incipient distress. The therapist then orients to the client's turn by seeking confirmation that her watching the video was responsible for her being caught off guard. After showing strong agreement—first through multiple head nods and then through a series of three consecutive *yeah*s—the client slightly expands on her turn by uttering “>well it < still does.” (line 12). The design of and interactional features surrounding this turn merit further discussion. First, the prefacing 2.7-s pause in line 11 may be signaling a hesitation to continue. Second, the “well” is indexing the response's *non-straightforwardness* (Schegloff and Lerner, 2009) and, further, that there may be much more to say. Third, her use of “still” does temporal work by extending her experience of “being caught off guard” to present time and, therefore, further underscores the relevance and impact this experience had and is *still* having on her.

In line 15, the therapist responds by focusing the temporal context of the client's experience more precisely on the present moment: “what's happening inside °right now.°” Questioning formats containing temporal markers, such as “now” or “right now” have been termed *immediacy questions* that guide client experience into the here and now (Kondratyuk and Perakyla, 2011). With this move, the therapist provides the client with an opportunity to elaborate more deeply on her present feelings with respect to the video. But rather than comply with the therapist's request and launch into emotion talk, the client instead utters an account that relates to her lack of ability and energy: “see I- I ↑don't have any good- < uh good resources right now >cause I'm < quite tired.” Eve's response is once again produced with a tremulous voice, thus indexing a form of distress. Her up/down dampening motion with her hand (lines 19–20) may also be acting as a “brake” against further attempts at exploring her present feelings. On the one hand, Eve's account reinforces the view that she is vulnerable and helpless; that is, it implies a diminished personal agency in which she may not be able to effectively deal with emotional issues. On the other hand, by adding “right now” to her account, she seems to also be resisting the *membership category* (Sacks, 1995a) of being a *vulnerable person*; that is, the vulnerability is not an enduring trait but is only applicable to her in this specific context.

The therapist then shifts her focus away from Eve's present feelings in relation to the video toward the feelings articulated in Eve's account. She does this by making two attempts at prompting the client to elaborate via a general elicitation in line 21 (“↑uh huh:?”) and a more specific elicitation in line 26 (“°what does that mean.°”)^2^. The client's repeated conduct of withholding from responding could be conveying opposition to the therapist's actions, but it may also be showing a form of “doing being upset.” For example, these client silences, coupled with a sigh in line 28 (“.hhh hhh”), seem also to convey a sustained level of distress. This then leads the therapist to produce a candidate answer that topicalizes and simultaneously seeks confirmation of the client's depressed state or helplessness and vulnerability: “you're feeling: [low?] >or you're feeling < vulnerable?” Eve's answer in lines 33 and 34 is produced in a *dispreferred* format (Pomerantz, 1984; Sacks, 1987) that does not grant confirmation of the therapist's candidate choices. First, the turn-initial “well” signals an upcoming non-straightforward answer; second, Eve's response orients to the second option posed in the therapist's question, rather than on her “feeling low,” by resisting the term “vulnerable” through her selection of the term “delicate” instead; and third, by stating “feeling more delicate *than normal*,” she implies that her delicacy is an exceptional case, thus again resisting the membership category of “delicate/vulnerable person” as an enduring trait.

**Extract 1**: 01:12–02:20^3^. 
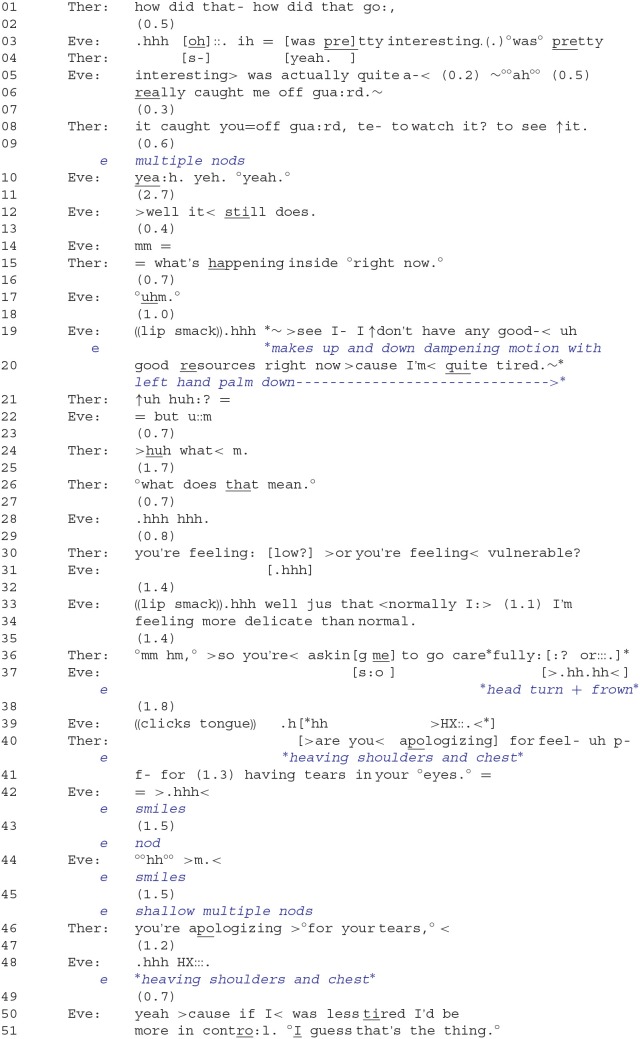


Dispositional claims, such as “not having good resources,” “being tired,” “feeling delicate,” etc. index the *inference rich* character of “vulnerability” (Sacks, 1995a, p. 40) and, further, open up certain possibilities for responding. In lines 36–41, we see the therapist specifically orienting to these aspects of the client's here-and-now experience by drawing two types of implications. To begin, the therapist formulates the upshot of “feeling vulnerable” in which the therapist is required to proceed gently and cautiously when responding to the client's distress: “>so you're < asking me to go carefully::? or:::.” The client, however, seems to reject this option in line 37 by making a negating head turn (on the horizontal axis) while frowning. The therapist then articulates another possible interpretation, which is that the client is apologizing for her display of a negative emotion (“having tears in your °eyes.°”) and receives non-verbal confirmation from Eve in lines 43 and 45. Eve also smiles in lines 42 and 44, which may be signaling admission or that she is “pleading guilty” to having displayed her emotions. This latter interpretation seems to bring the focus of talk back toward Eve's initial displays of distress; that is, her tearfulness may be linked to what she had felt when watching the video. Then, in line 46, the therapist redesigns the latter reading of the client's implied action as a formulation (“you're apologizing >°for your tears,° < ”), thereby seeking more explicit confirmation from the client. Following a 1.2-s pause and a pronounced sigh that in turn-initial position projects an upcoming dispreferred response (Hoey, 2014), the client once again provides an account. But this time, she lists “having less control” as a reason for not being able to manage or restrain her emotions. Here again, the client depicts herself as not operating at “full capacity,” and the inference may be drawn that the client is at risk of being vulnerable (i.e., she may be susceptible to intensely experiencing her distress), somewhat helpless (i.e., she may not be able to control the emotions associated with her distress), and thus not ready to confront her present emotions head on.

### Phase II: Managing Continued Opposition to Emotional Exploration

The prior extract has shown that the brother's death is a locus of distress for the client and thus constitutes a relevant theme in therapy. Focusing on the client's distress displayed in the moment by an immediacy question did not, however, result in the further exploration of the client's present emotions concerning the brother. This may be because clients who are experiencing deep distress involving painful past events may be reluctant to express their emotions with more intensity or may not be ready to engage in conversations that explore or interpret their grief. As argued by Greenberg et al. (1993, p. 274), “for most people in therapy there is some sense of vulnerability, embarrassment, or shame in revealing their most personal and vulnerable aspects. There is a sense of risk in sharing experiences that are uncomfortable and private.” During this phase of interaction, the therapist would respond to the “content” of the client's distress talk. Thus, by orienting to what the client is saying in her reporting of distress rather than what she is currently feeling, there becomes much less pressure for the client to engage with her emotions in the present. The client, Eve, however, would tend to reject and criticize therapist formulations that worked to explore the content of Eve's reported upset, and this led the therapist to topicalize the therapist–client relationship and Eve's negative emotions directed toward the therapist.

#### Focusing on the Reported Aspect of Distress: Circumventing the Client's Vulnerability

It was found that when the therapist stayed relatively close to the client's own words, as for example by formulating the gist of client's prior talk, the client would tend to offer agreement and affiliation with the therapist's action. However, when the therapist attempted instead to point out relevant implications of the prior talk, the client would not only voice her disagreement but would also mock or criticize the therapist as having responded in an inappropriate fashion^4^. Consider Extract 2.

In lines 01–05, Eve uses expressions, such as “ho::w, (2.2) deep my feelings we:re” and “ho:w, (0.9) pro ↑ foundly. (2.1) it affected the course of my li:fe.” to report on the significance her brother's death had on her. Furthermore, she frames these significant aspects in terms of not having known this beforehand and, thus, as a revelation (i.e., “I: had no idea”). Eve's tremulous voice, interspersed with affect-laden sighs (lines 02 and 10), seems to display severe distress at gaining this newfound knowledge. What begins to emerge here also is Eve's portrayal of herself as vulnerable to unforeseen events happening in her life, events that she does not seem to have any control over. The therapist briefly responds by first offering minimal affiliation with Eve's affectual stance of distress through a head nod in line 06 and then by producing a continuer that prompts more talk from Eve. In lines 08–14, the client elaborates on how her life had been affected: The first expression, “cruising o:n.,” implies a carefree and unconstrained attitude; the second, “scra:mbling,” is more negative, relating to life being lived in a frantic, confused, and disorganized manner; and the third, “treading wa:ter.,” implies a standstill and that there is no progression or development happening for the client.

By way of response, the therapist initially displays empathy with the client's reported distress by formulating the gist of the client's message, in a way that subtly transforms yet stays close to her wording. For example, the client's “I: had no idea” becomes rephrased as “you:: hadn't really fully:> appreciated” and “I > was just< like, (0.3) cruising o:n” as “you were kind of-° (0.4) tryin to carry on blithely.” Furthermore, the therapist's metaphorical expression “maybe there's a hole in your ship” (line 22) offers a relevant extension in meaning to the client's use of “scrambling” and “treading water,” for it also implies vulnerability; that is, a hole may cause a ship to sink. It should be noted that the client conveys affiliation along many points of the therapist's turn and afterwards. Eve consistently nods during and immediately subsequent to the formulation and verbalizes agreement in line 26 (see Stivers, 2008; Muntigl et al., 2012).

**Extract 2**: 05:31–07:42. 
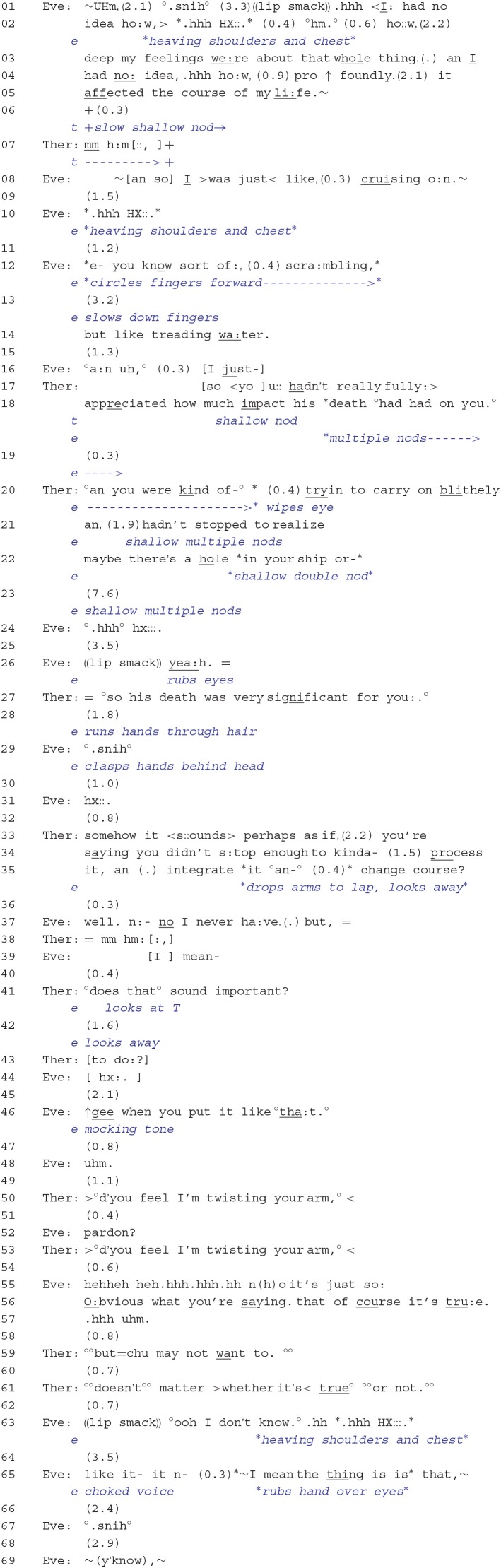


It is at this point, however, where the conversation proceeds to get off-track. Starting from line 27, the therapist initiates a shift in frame in which she begins to move away from the client's initial revelation and the ways in which her life had been affected into an activity that focuses on the implications of the client's talk (“°so his death was very significant for you:.°”). Furthermore, the client withholds her confirmation from line 28, which may be conveying implicit disaffiliation or even that she is having a hard time grasping the impact that her brother's death had on her life. In line 33 onwards, the therapist continues to draw implications, but prefaces her turn with the epistemic markers “it < s::ounds>,” “somehow,” and “perhaps.” In this context, where the client has not displayed explicit affiliation in her prior turn, the therapist seems to be orienting to this ascription as being something delicate to do. The therapist's formulation explicitly points out the possible consequences of not having “fully appreciated” or “realized” the impact that the brother's death had on her; that is, Eve may have taken more time to reflect on these events (“process it, an (.) integrate it”) and to take a more agentive role in her life (“change course”). This more interpretive move by the therapist may be seen by the client as no longer fully endorsing her original stance and that may explain why, in line 37, Eve starts her turn by reluctantly agreeing with the therapist (“well. n:- no I never ha:ve.”) and then produces a disagreement token “but.” The therapist then takes another turn (line 41) that explicitly seeks confirmation from the client (“°does that° sound important?”), but rather than offer her endorsement, the client continues to disaffiliate by turning away (line 42) and by producing an exasperated outbreath that overlaps with the therapist's turn continuation (“to do:?”).

Explicit disaffiliation occurs in line 46 when the client underscores the “obviousness” of the answer while simultaneously mocking the therapist (“↑gee when you put it like °tha:t.°”). It is here that the client's dilemma in reference to the problem of experience becomes apparent: she is being confronted with an expert's view and (rational) understanding that this perspective on her is correct, which, at the same time, does not orient to her in-the-moment experience of feeling devastated, vulnerable, and exposed. Thus, the “mocking tone” would be indexing the client's reluctance to get in touch with her feelings, but she *is* admitting that the therapist has made a point. Furthermore, Eve's derision is doing additional emotional work; for example, by mocking the therapist, Eve seems to be conveying her annoyance with what the therapist has said and, by implication, that she may be displeased or angry with the therapist^5^.

After withholding from taking up a turn at talk and thus allowing the client to continue and account for her disaffiliative response (lines 47–49), the therapist then orients to the interactional trouble by suggesting a possible reason for the client's displeasure (“>°d'you feel I'm twisting your arm,° <”); that is, the client may feel that the therapist's interpretation was made too forcefully and is perhaps not in step with the client's own perspective. The therapist's response also orients to who has primary rights to control the direction of the interaction, termed *deontic status* (Stevanovic and Peräkylä, 2014), suggesting that the therapist may have overstepped her bounds. Following a brief *other-initiated repair* sequence in lines 52 and 53 (Schegloff et al., 1977), the client first denies the therapist's reason and then provides an account that criticizes the therapist's prior intervention as being incongruous and inappropriate and challenges its relevance (“it's just so: O:bvious what you're saying. that of course it's tru:e.”). There may also be an implication of a breach in the therapist's genuineness or authenticity (Rogers, 1957); that is, in stating the “obvious,” the therapist may be running the risk of appearing as lacking an adequate professional commitment and as simply supplying formulaic expressions as a response to the client's troubles. This criticism conveys an affectual stance of continued anger or annoyance at the therapist's response, but what also seems to surface from this is the mismatch between what the client is emotionally experiencing, on the one hand, and the therapist's attempts at describing and exploring her distress, *through words*. This discrepancy will resurface again later and become highly salient in Extract 4.

Subsequently, the therapist does further work to re-establish affiliation, agreement, and a shared perspective on the prior interactional trouble. In line 59, the therapist's utterance (“^°°^but = chu may not want to.^°°^”) displays her understanding of the client's prior disaffiliative action of line 37; that is, although the client may have realized that she could have more deeply reflected on and dealt with the brother's death, she has no desire to do so. But following “no response” and thus “no confirmation” from the client in line 60, the therapist then provides another opportunity to engage the client by orienting to the implication in the client's prior turn that the therapist's interpretation is not relevant (“^°°^doesn't^°°^ matter >whether it's < true^°°°^or not.^°°^”). However, even this attempt fails to garner an affiliative response. Rather, the client first makes a claim of no knowledge (“°ooh I don't know.°”), which seems to simply dismiss and frustrate the therapist's line of action (Drew, 1992; Hutchby, 2002), then produces a prolonged sigh, and finally proceeds to return to the topic of the brother.

#### Topicalizing the Relationship: Eve's Anger Toward the Therapist

Within this phase of the therapy, there were repeated sequentially unfolding cycles in which the client reported on and displayed her distress, followed by the therapist's formulation of Eve's experience, ending with Eve criticizing or reprimanding the therapist. Psychotherapy researchers have referred to such episodes as alliance ruptures, especially in relation to strains in the therapist–client relationship, and one of the suggested ways in dealing with these ruptures is to *explore relational themes associated with the rupture* (Safran et al., 2011). For example, clients may feel resentful that the therapist is intruding in the client's personal experiential domain and therapists may thus respond by addressing the client's feelings toward the therapist. Consider Extract 3. Just before this stretch of talk, the client and therapist jointly produced sequences in which the client reported on her distress and on the deep significance of the video: “it's like, (0.9) the la:st 2 weeks have jus- (0.7) not >existed <”; “it's like anything that I was doing in my life is just a shadow.”

In lines 01–05, Eve uses vivid descriptors to characterize the issue with her now deceased brother and his wife (i.e., Kevin and Jennifer) as “fresh and ra:w.” and conveys distress throughout her turn by repeatedly sighing and adopting a wobbly voice. The therapist, in line 09, produces a gist formulation that captures the metaphorical dimension of Eve's talk (“an open so:re”), which then receives confirmation from the client. Eve continues by stating that this experience prevents her from being rational and disciplined but then ends her turn in line 14 with “I- I >don't °know what I'm saying.° <,” while conveying distress by covering her eyes with her fingers, rubbing her eyes, and producing an intense sigh. Here, Eve is expressing her inability to continue, but she is also pointing to the difficulty in articulating her feelings and, by implication, may not find it appropriate for the therapist to continue with talk that is focused on her experience. The therapist, however, responds with a formulation that directly engages with Eve's prior talk. She orients to the intensity of her experience (“so <bi:g”; “kin've = jus seeps into everything”) and how this may be making it difficult for her to be rational. In line 21, the therapist returns to the topic of Eve's “fee:ling very emotional,” but then does not complete her utterance. Eve responds with an “°I dunno.°” in line 23, which disaffiliates with, and thereby resists, the therapist's line of exploration (Drew, 1992; Hutchby, 2002), and then makes explicit distress displays by rubbing her eyes, sighing, and holding her fingers at her temples while gazing downwards. By way of response, the therapist now shifts the focus of the conversation by orienting to what Eve's distress might be conveying at the relationship level; that is, Eve's difficulty in engaging with and endorsing the therapist's responses may have to do with feeling resentment at having to watch the video the previous week and, therefore, being angry toward the therapist and research group (lines 27–31). Eve, however, strongly resists this interpretation and begins to repeat how “hard” and “intense” her experience was.

**Extract 3**: [10:51–12:20]. 
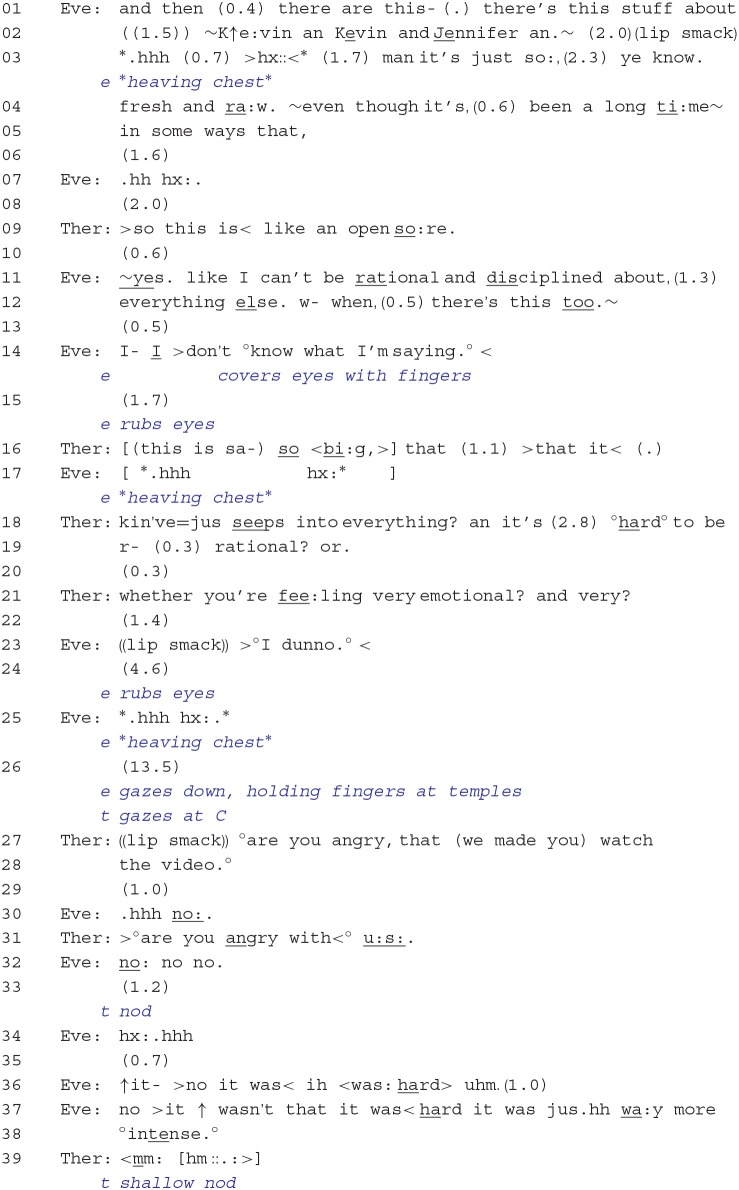


### Phase III: Successful Guidance Into Emotional Exploration

The therapist's attempts at getting the client to focus on her felt emotions in the present moment and at exploring her reports of distressing experience have thus far not received much affiliative uptake by the client. Around 15 min into the session, however, a noticeable shift happens: to begin, the therapist frequently punctuates the interactional sequences with actions that guide the client into the immediacy of her emotional distress. By placing or “timing” her responses in this way, the therapist was able to facilitate a very different trajectory in which the client displayed her distress in repeated sobbing episodes. Furthermore, the problem of experience in terms of the client's difficulty in talking about her distress (because of her vulnerability, feeling devastated, and lacking control) and the therapist's attempts at providing a “rational-empathic” interpretation of Eve's distress becomes resolved. Once Eve's emotions “flood out” or become intensely displayed in the moment, the therapist helps to maintain a high degree of emotional intensity through directive actions.

#### Overcoming Vulnerability: Guiding Client Into More Intense in-the-moment Emotional Work

The progression in which the client moves toward engaging in more intense emotional work in the present moment is shown in Extract 4. As in previous extracts, the therapist's display of understanding through upshot formulations was consistently rejected by the client.

The very beginning of this exchange, lines 1–19, follows a sequential pattern that bears much similarity to Extract 2. The therapist provides an upshot formulation that seeks confirmation about the centrality of Eve's feelings toward her brother and his wife. Following hesitation, silence, and expressions of uncertainty from Eve (lines 04–07), the therapist produces a question that can be interpreted as targeting the client's *in situ* emotional state (“what's happening”) (Kondratyuk and Perakyla, 2011). This leads Eve in lines 12–16 to develop an elaborate emotional stance of anger in which she ridicules the therapist's earlier attempt at getting Eve to focus on “what is central” for her. Here again, as in Extract 2, the client is berating the therapist for having produced talk that is too rational in its focus and that does not match Eve's present experience of the distressing event. The therapist thereafter attempts to reaffiliate with the client by echoing her criticism that the formulation was too rational in scope (“that sounds too rational?”) and was incongruous with Eve's feelings (“it doesn't. fit somehow?”).

But now, rather than allow Eve to continue with narratives topically related to her brother—as she did in Extract 2—the therapist produces an immediacy question that provides the client with an opportunity to explore what she presently feels (“what is going on.”) and, thus, to provide the kind of talk that may “fit” with her experience. What ensues is a sequence comparable to Extract 1: the client initially avoids answering the question through a prefacing 1.2-s pause and an “I >dunno. < ,” followed by an account that makes an appeal to her momentary vulnerability (“I'm feeling tired? I'm a little delicate”). At this point, Eve also begins to reveal signs of distress, as shown by her tremulous voice and her pronounced turn-final sigh (Hoey, 2014). The therapist then orients to the client's opposition to probe her own present feelings more deeply by offering the client affectual terms that more strongly index “hurt” and “vulnerability” (“you're feeling < bruised>? … fragile?”). But instead of continuing to make her delicacy or vulnerability a topic of the conversation, Eve reframes the impasse to exploring her present experience by recycling the “rationality argument” made previously; that is, in terms of her emotional experience regarding her brother's death, she claims that “~it's like it's too: emotional ta=even talk about.~” and, a few lines down, states that words cannot adequately express what she feels. The implications for the ensuing client/therapist interaction are as follows: First, the client's turn tends to discourage further formulations or interpretations of the client's current feelings, and second, she may be signaling a need to explore her feelings at the level of “emotional displays” rather than through talk. Thus, the client's turn may be seen as an invitation to the therapist to help facilitate this line of activity.

What then follows is a carefully orchestrated and negotiated interactional sequence in which the client is able to express her emotions in relation to her past experience of having watched the video. To begin, the therapist leans in toward the client at line 35, creating less physical distance between them. Through this bodily movement, the therapist creates the possibility for more intimacy and a more secure space in which to be delicate or vulnerable. Then, following a long 6.5-s pause, the therapist rephrases the client's prior talk by emphasizing the mismatch between words and feelings *in the present moment* (“doesn't >seem as though< <wo:rds,> (0.4) can express for you. (0.4) what you're feeling °right now,°”) and by implying that emotion work can only now be accomplished through *in situ* emotional experiencing. During the latter part of the therapist's turn, the client begins to shake her head in agreement and, in the subsequent silence, brings her hands to her eyes and then begins to weep. The therapist in line 48 continues on with her turn by drawing the client even further into the present moment. She does this by producing a *noticing* (Schegloff, 1988; Muntigl and Horvath, 2014a) that draws attention to her emotional display (“°but you did yourself just° feel it there?”). With this action, it is implied that although words may be an insufficient means to talk about her feelings, that no longer need concern them because she is now able to connect with her feelings without “words.” Furthermore, the use of “just” emphasizes the client's here-and-now experiencing, drawing attention to the client's immediate display of emotion. Following this, the client seems to physically shield herself from the therapist by completely covering her hands with her face and then weeps for several minutes, as represented by a long series of in- and out-breaths (Hepburn and Bolden, 2017), before taking up another turn at talk (lines 50–53).

**Extract 4**: 13:11–16:08. 
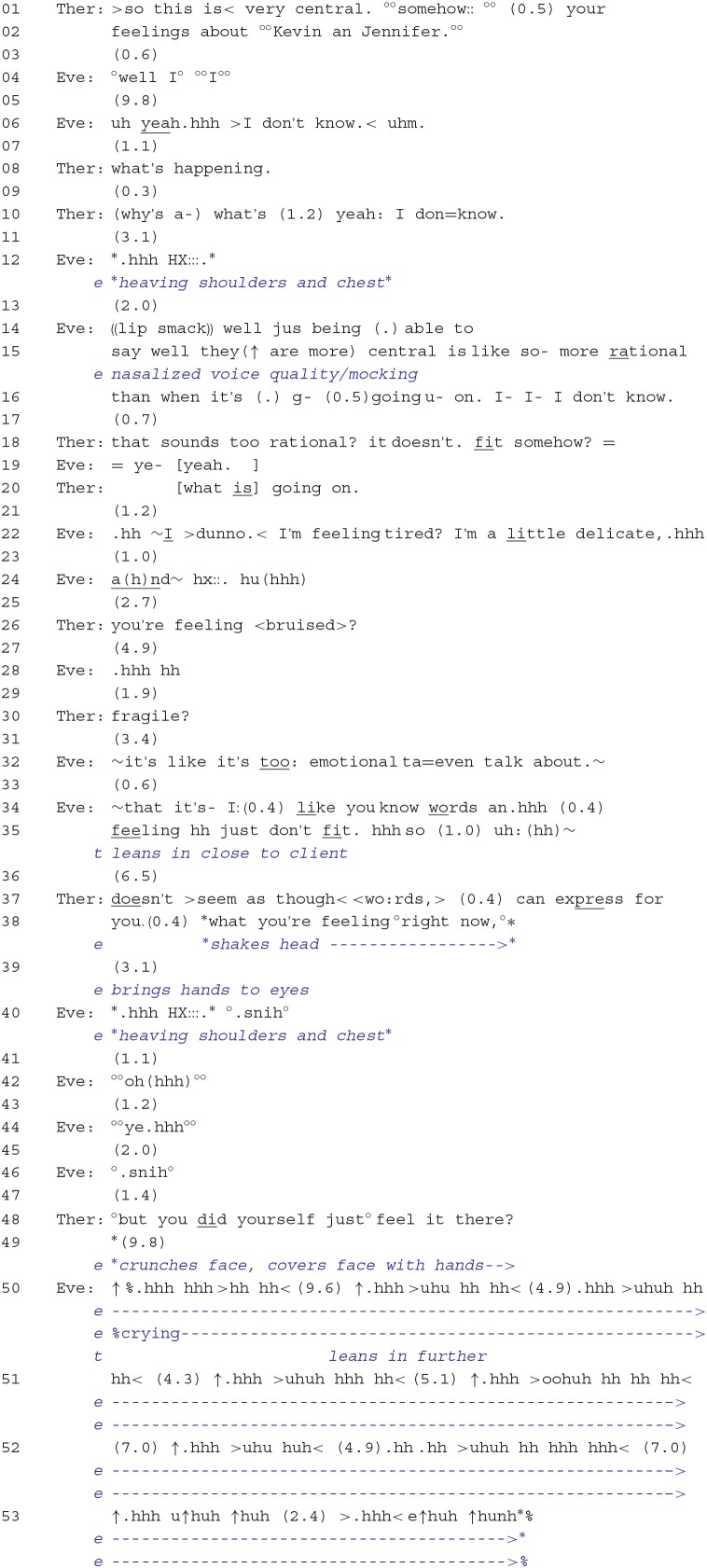


Thus, it would appear that the therapist's placement and timing of her interventions played a crucial role in getting Eve's emotional outburst underway: By offering Eve a secure space to experience intense emotions, by repeatedly drawing the focus of talk on the client's presently felt emotions, and by openly conceding that Eve's distress should be explored by experiencing it in the moment (rather than talking about it “rationally”) led to a joint understanding of how emotional exploration could effectively proceed (i.e., an empathic moment) and, thus, to the client's readiness to engage in intense emotional work.

#### Maintaining the Client's Focus on Her Distress

The next 10 min of interaction primarily involved prolonged episodes of sobbing, and these episodes were interspersed with brief interaction sequences in which the focus of talk was placed on the client's emotional distress, followed by therapist practices that guided the client back into experiencing and displaying her immediate distress. Consider Extract 5.

During Eve's assertion that she just wants to “cry an cry an cry an cry,” the therapist offers affiliation through nodding and then by responding in line 06 with acknowledgment (“mm hm:”) and a gist formulation that underscores Eve's need to express her sadness in the present moment (i.e., *right now*). The therapist's utterance in line 11, “there'll be time °for <words.>°,” implies that the exploration of the client's distress through talk should take a back seat to the importance of having Eve express her emotions. Following a 6.1-s pause, the therapist then directs the client to weep using an imperative (grammatical) format (“(>so you<) let it out.”). The imperative design of this directive indexes low contingency (e.g., there is no use of modal expressions, such as *could you* to cater to the client's ability or willingness to perform the action) and the therapist's high entitlement to perform the directive (Curl and Drew, 2008; Antaki and Kent, 2012; Drew and Couper-Kuhlen, 2014). The client's immediate compliance, shown by her engaging in another extended sobbing episode, attests not only to this ratified role relationship but also to the continued secure environment enabling her to weep in the therapist's presence.

## Discussion

Research has already demonstrated that, to achieve an elaborate understanding of how therapeutic projects actually unfold over time, it is important to examine longer stretches of interaction, to ascertain whether certain therapeutic interventions are functioning in a more (or less) productive way (e.g., Voutilainen et al., 2011; Muntigl, 2013; Buchholz and Kächele, 2017). This paper has shown that a client's distress may need to be managed over many sequences and that the ways in which distress is dealt with in one interactional phase may occasion different responses from the therapist. But unlike some institutional activities, such as problem presentation and information gathering during primary care visits (Robinson, 2013) or soliciting chair work entry in emotion-focused therapy (Muntigl et al., 2017), which seem to be strongly goal-directed and follow distinct interactional patterns, the direction that the activity of managing distress will take seems to be strongly contingent on the therapist's ability to maneuver around client opposition and deal effectively with the client's intense upset, rather than adhering to goals as such.

There is, however, a model of psychotherapeutic development, originating from the Mount Zion Group in San Francisco (Horowitz et al., 1975; Gazzillo et al., 2019) that bears similarity to the kinds of interdependent, interactional phases being proposed here. Briefly put, these authors state that only after the therapist has successfully passed an interpersonal challenge from the client will clients disclose previously avoided distressing experiences. The claim is that clients will present the therapist with various forms of tests or challenges, such as disagreeing or being angry with the therapist, to gauge the degree of safety on hand (Horowitz et al., 1975). Thus, if therapists are able to “pass the test” by dealing effectively with clients' disagreement or anger, clients will feel secure enough to disclose their distressing experiences. Comparing the above model with this examination of client-centered therapy, the attention was placed on the challenges that the therapist was faced with when responding to client reports or displays of distress. This “problem of experience,” as coined by Heritage (2011), was especially salient when the therapist formulated certain implications arising from the client's reported experience. This was taken as inappropriate and was severely criticized, implying that the therapist's understanding of the client's grief was incorrect and unfitting (i.e., too rational). These difficulties in offering “suitable” empathy also seemed to generate implications for what Rogers (1957) had termed being genuine or authentic. By stating the obvious and by providing “rational understandings,” it was implied that the therapist cannot fully grasp what is at stake for the client and that the therapist must therefore revert to formulaic expressions. This led to talk in which the therapist topicalized a rupture in the alliance or the growing strains being placed on the therapeutic relationship (Safran et al., 2011).

**Extract 5**: 20:20–21:24. 
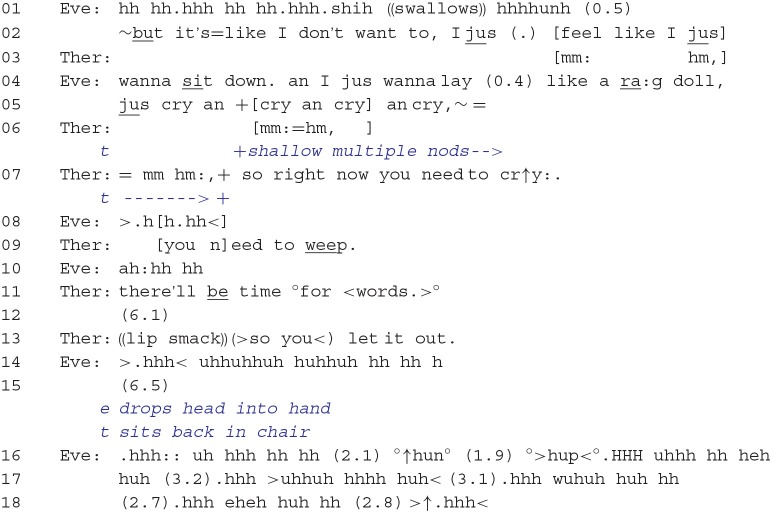


The overall trajectory of this session of client-centered therapy showed, however, that the therapist and client were eventually able to overcome impasses (in the form of challenges or tests) to exploring the client's present emotions more deeply, and it would seem that affiliation and safety were key factors in enabling this outcome^6^. First, pertaining to social solidarity, the client's eventual engagement with her feelings in the here and now seemed to index an empathic moment, in which shared understanding of how to deal with the distress and mutual affiliation could be realized (Elliott et al., 2011; Heritage, 2011). Finally, weeping has been argued to index intense sorrow and helplessness and suggests that the person has “surrendered” and abandoned all efforts at coping (Frijda, 1986). Eve's extended bout of weeping, therefore, seems to point to the establishment of a secure relationship, partially facilitated through the therapist's bodily action of decreasing the space between her and the client, in which she can *be vulnerable* in the presence of another. It may thus be said that, although there does not seem to be any “hard” interactional evidence that the client was in fact testing the therapist through her repeated opposition or disaffiliation and her displays of annoyance or anger, it does appear, as Horowitz et al. (1975) are suggesting, that establishing client safety is important for moving beyond impasses occurring in therapy.

What this examination has also shown are some of the ways in which a therapist may orient to the client's distress. Drawing from the distinction of Antaki et al. (2015) between *reporting* vs. *in-the-moment distress*, it was shown that the therapist would orient to the former by formulating the client's personal experience. In-the-moment distress, by contrast, was oriented to not only via *immediacy questions* (“what's happening inside °right now.°”) and *noticings* (“°but you did yourself just° feel it there?”) but also by directive actions that guide clients into the re-entry of a sobbing episode [“(>so you <) let it out.”]^7^. It may also be said that these response types to distress are not random choices but are predicated on the immediately prior context or on the kind of interactional work accomplished in a phase of talk, often involving client opposition or the therapist being challenged. Orientation to the client's reporting of the distress, for example, occurred only after the client had resisted exploring her in-the-moment distress^8^. This therapist also first began to manage the client's distress through an immediacy question, thus orienting first to the client's here-and-now distress display and later used a noticing to guide the client into a deeper form of emotional expression. Finally, directive actions were used only when the client had already accomplished prolonged in-the-moment emotional work. Thus, this study sheds light on the context sensitivity of orienting to distress and that certain practices may be uniquely shaped by what had occurred in prior talk.

There are certain limitations to this study. Only one session involving one therapist–client dyad was examined. Future studies, drawing from a larger corpora of distress display sequence trajectories with more clients and therapists of varying therapeutic orientations, will be needed to extend our understanding of the diversity in which episodes of upset may be responded to and managed. What has been shown, however, is that longer-term sequential trajectories may be fruitfully analyzed by focusing on a specific sequence type (i.e., distress display + response) and its reoccurrence over time and that, in doing so, a certain distress management trajectory comes into view—compare similar longitudinal studies that focus on Question/Answer or Conclusion/Response sequences to track resistance over time (Voutilainen et al., 2011; Muntigl, 2013). This study has highlighted the challenges that clients and therapists face when clients are confronted with distressing personal experiences. Horowitz et al. (1975) have claimed that, to move forward, clients need to challenge or test therapists to gain reassurance that it is safe to explore their distress. This paper has illustrated how a secure relationship, one that facilitates therapeutic work, may be accomplished interactionally. Three main points may be mentioned in conclusion. First, productive rupture and repair sequences involving weeping in client-centered psychotherapy may index a change process. Second, using a more content-focused approach (e.g., via formulations) in response to strong client emotional pain or client opposition may be insufficient and can lead to therapeutic impasses or ruptures. Third, a more process-focused approach to emotionally laden client experiences can be more effective and can facilitate extended and productive client emotional expression.

## Data Availability Statement

All datasets generated for this study are included in the article.

## Ethics Statement

The study involving human participants was reviewed and approved by Simon Fraser University Research Ethics [2012s0672]. Written informed consent was obtained from the participant to participate in the York I study and for the publication of anonymized data.

## Author Contributions

PM performed the analysis of the extracts and wrote the full paper.

### Conflict of Interest

The author declares that the research was conducted in the absence of any commercial or financial relationships that could be construed as a potential conflict of interest.

## Footnotes

^1^Other formulation types have been identified by Weiste and Peräkylä (2013) and are termed *relocating* and *exaggerating*, but these seem to be restricted to psychoanalysis and cognitive psychotherapy.

^2^See Muntigl and Zabala (2008) for a discussion of general vs. specific elicitation practices in psychotherapy.

^3^“01:12–02:20” refers to “time into the recorded session.”

^4^This sequential progression of moving from summaries or “reflections” of client's talk to drawing implications or interpretations of this talk may be a general practice that occurs in different therapy approaches, including cognitive-constructivist psychotherapy (Voutilainen et al., 2010). Through this practice, the therapist first secures client endorsement before exploring various implications, consequences, or perspectives (stemming from the client's initial report) that the client had perhaps not considered.

^5^Reviewer 1 has pointed out that, in emotion-focused therapy terms, the client's anger at the therapist could be construed as “secondary reactive” anger, which may be covering up more basic feelings of psychological pain and/or guilt and may also have been used to “interrupt” the psychological pain.

^6^Outcome measures have classified this client as recovered from depression at the end of therapy. Although there is no direct evidence that the analyzed trajectory was mainly responsible for this outcome, it is conceivable that the identified shifts in emotion talk played a role in this development.

^7^It should be noted that emotion-focused therapy researchers have identified a list of therapist response types that bear resemblance to the action types of CA (see Elliott et al., 2004). They use speech act labels termed Experiential Response Modes to characterize the different kind of empathic vs. process-oriented actions that therapist responses may be performing. Further research is needed to explore how CA action terms and Experiential Response Modes may complement each other.

^8^There is another possible reason to explain the therapist's initial use of *empathic-oriented* (e.g., formulations) rather than *process-oriented* (e.g., noticing) responses (Elliott et al., 2004). From research protocol of the York 1 study, which offered either client-centered or emotion-focused therapy to clients, therapists were discouraged from using process-guiding interventions in the client-centered condition. Thus, the therapist here might have initially held off from responding to the client's distress in a more processing guiding, less “rational” manner, thus generating the therapeutic rupture that eventually led the therapist to change tactics and shift to a more flexible way of working with the client. I thank Reviewer 1 for having suggested this.
